# Supplementary material for: Graph embeddings for identifying symmetries in Connectomes
Source: bioRxiv. 2025 Dec 11:2025.12.06.692615. Preprint. [Version 2] doi: 10.64898/2025.12.06.692615 (PMC12709479; doi:10.64898/2025.12.06.692615)
Supplement: Supplement 1 [file NIHPP2025.12.06.692615v2-supplement-1.pdf]

bioRxiv preprint doi: https://doi.org/10.64898/2025.12.06.692615; this version posted December 11, 2025. The copyright holder for this preprint (which was not certified by peer review) is the author/funder, who has granted bioRxiv a license to display the preprint in perpetuity. It is made available under aCC-BY 4.0 International license.

6 Appendix

6.1 Cell types considered to be in the central complex

We manually selected a subset of all cell types in the hemibrain dataset and considered these to be the "central complex" cells, based on the list in Scheffer et al. (2020), Appendix 1 Table 5. Note that cell types with fewer than 10 cells are always removed. The 66 types that remain are Delta7, EL, EPG, FB4E, FB4Z, FB5V, FC1A, FC1B, FC1C, FC1D, FC1E, FC1F, FC2A, FC2B, FC2C, FC3, FR1, FR2, FS1A, FS1B, FS2, FS3, FS4A, FS4B, FS4C, IbSpsP, PEG, PEN a(PEN1), PEN b(PEN2), PFGs, PFL1, PFL2, PFL3, PFNa, PFNd, PFNm a, PFNm b, PFNp a, PFNp b, PFNp c, PFNp d, PFNp e, PFNv, PFR a, PFR b, hDeltaA,

bioRxiv preprint doi: https://doi.org/10.64898/2025.12.06.692615; this version posted December 11, 2025. The copyright holder for this preprint (which was not certified by peer review) is the author/funder, who has granted bioRxiv a license to display the preprint in perpetuity. It is made available under aCC-BY 4.0 International license.

hDeltaB, hDeltaC, hDeltaI, hDeltaJ, hDeltaK, hDeltaL, vDeltaA_a, vDeltaA_b, vDeltaB, vDeltaC, vDeltaD, vDeltaE, vDeltaF, vDeltaG, vDeltaH, vDeltaI, vDeltaJ, vDeltaK, vDeltaL, and vDeltaM.

6.2 Connectivity tuning curves

Let $\boldsymbol{W}_{\text{sorted}}$ be the connectivity matrix, sorted by the estimated phases of neurons in each cell type (i.e., Fig. 2B). Let $W^{A,B}_{\text{sorted}}$ be a submatrix showing the weights from type A neurons and type B neurons. Then the A-to-B-to-A effective weights are defined as $W^{A,B}_{\text{sorted}}W^{B,A}_{\text{sorted}}$ and similarly for A-to-B-to-C-to-A. As long as the chain begins and ends with type A, the result is a square matrix with rows and columns sorted by the estimated phases of type A neurons. The effective weights are normalized such that the maximum element is 1.

6.3 Phase-shifted connections

If we label EPG neurons with evenly spaced angles $\theta^{\text{EPG}}_k, k = 1, ..., N^{\text{EPG}}$ and PEN neurons with $\theta^{\text{PEN}}_{k'}, k' = 1, ..., N^{\text{PEN}}$, we can write the EPG-to-PEN weights as a function of $|\theta^{\text{EPG}}_k - \theta^{\text{PEN}}_{k'}|$. However, the PEN-to-EPG weights are "shifted" in that they are given by a function of $|\theta^{\text{EPG}}_k - \theta^{\text{PEN}}_{k'} + s_{\text{PEN,EPG}}|$, where $s_{\text{PEN,EPG}}$ is the phase shift (schematized in Fig. 5A). We also note that this structure need not be between only two cell types and can be informally stated as the following: For cell types $m = 1, 2, ..., M$, assign neurons from each type angles that are evenly distributed on the circle, $\theta^{(m)}_{k=1,...,N^{(m)}}$ and suppose that the connectivity from any type m to another type n is predicted by a function of $|\theta^{(m)}_k - \theta^{(n)}_{k'} + s_{m,n} + s_m + s_n|$, where $\{s_{m,n}\}, \{s_m\}$ are scalar phases. A "phase shift structure" occurs when it is impossible to find a set of "absolute" phases $\{s_m\}_{m=1,...,M}$ such that $\forall m, n : s_{m,n} = 0$.

How might this structure be detected in a large connectome efficiently? We first make the following observation. For simplicity, suppose type A and type B neurons are equally numerous and respectively embedded on two circles. This implies that each neuron is assigned an angle (Fig. 5B). Suppose we assign angles to the neurons such that the type A neuron with angle θ^{A}_k is connected to the type B neuron with angle $\theta^{\text{B}}_{k'}$, this implies that the two circles are aligned phase-wise (Fig. 5B, left). However, the type B→A weights suggest a different phase alignment, since the type A neuron with angle θ^{A}_k is now connected with the type B neuron with angle $\theta^{\text{B}}_{k'+1 \mod N^{(m)}}$ instead. In the embedding space, this means a relative rotation between the two circles, $\boldsymbol{Z}^A$ and $\boldsymbol{Z}^B$ (Fig. 5B). Thus, the phase shift structure between two cell types is reflected by a "tension" in the learned embeddings of these cells – the embeddings that best fit A→B weights and those that best fit B→A weights differ by a relative rotation between $\boldsymbol{Z}^A$ and $\boldsymbol{Z}^B$. Again, this can be stated in generality for multiple cell types. Suppose $\boldsymbol{Z}^{m=1,...,M}$ are learned embeddings of M cell types. A phase shift structure occurs when connections from type m to type n are well predicted by $\|\boldsymbol{R}_m\boldsymbol{Z}^{(m)}_i - \boldsymbol{R}_{mn}\boldsymbol{R}_n\boldsymbol{Z}^{(n)}_j\|$, where $\{\boldsymbol{R}_m\}$ and $\{\boldsymbol{R}_{mn}\}$ are rotation matrices. Then a phase shift structure occurs when it is impossible to find $\{\boldsymbol{R}_m\}$ such that $\boldsymbol{R}_{mn} = \mathbb{I}$ for all m, n.

To numerically learn $\boldsymbol{R}_{mn}$ using gradient descent on the loss function (Eq. 2), we parameterize it as $\boldsymbol{R}_{c_i,c_j} = \exp(\tilde{\boldsymbol{R}}_{c_i,c_j})$, where exp is the matrix exponential and each $\tilde{\boldsymbol{R}}_{c_i,c_j}$ is a

bioRxiv preprint doi: https://doi.org/10.64898/2025.12.06.692615; this version posted December 11, 2025. The copyright holder for this preprint (which was not certified by peer review) is the author/funder, who has granted bioRxiv a license to display the preprint in perpetuity. It is made available under aCC-BY 4.0 International license.

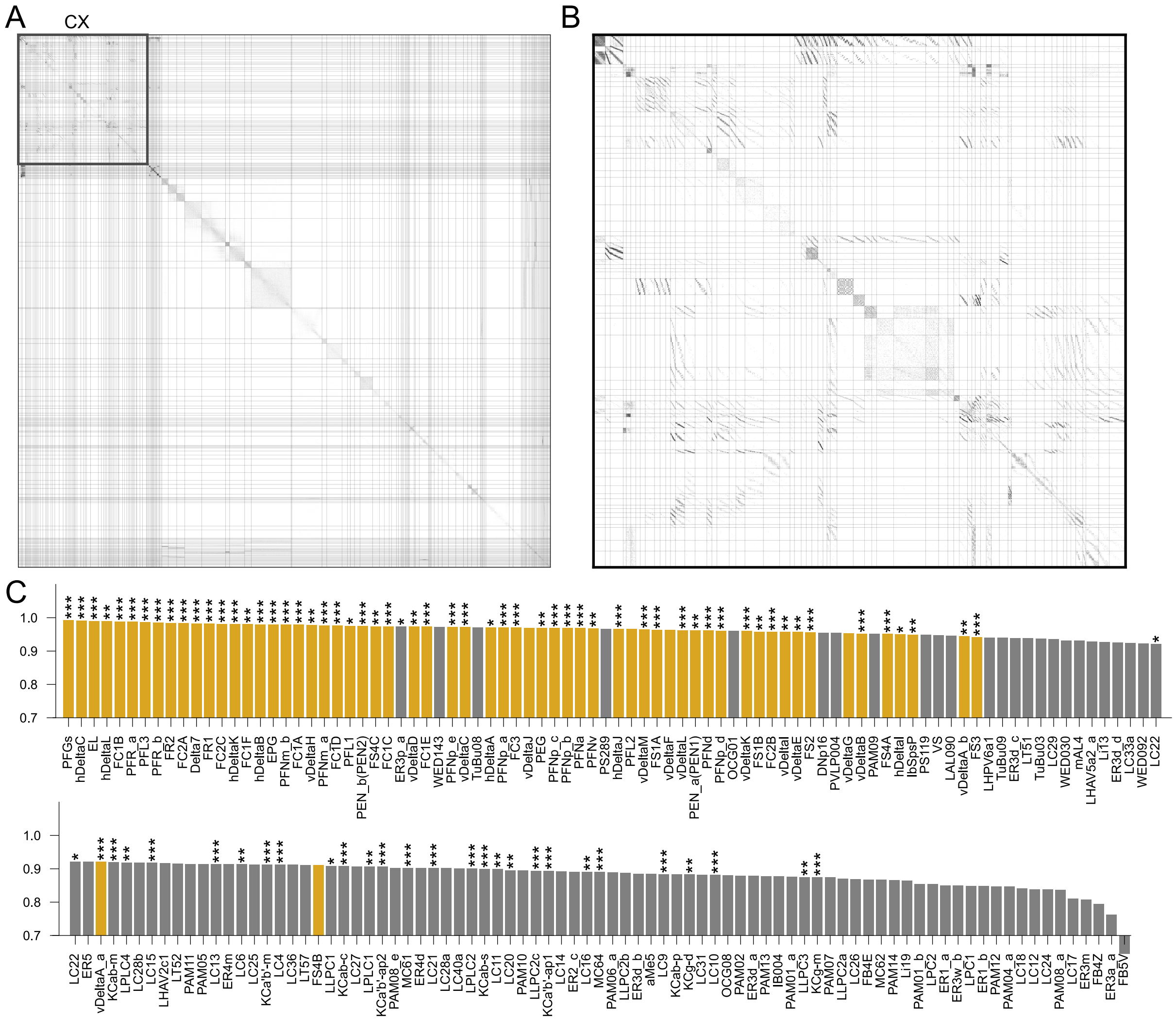

Figure S1: Embedding and sorting the entire hemibrain connectome simultaneously.
A Sorted connectivity in the whole hemibrain connectome.
B The section with connectivity within the central complex (CX), enlarged for details. Compare with Fig. 4A.
C Circularity coefficient of cell types in the entire connectome. Same as Fig. 4E but with labels. $^{*}p < 0.05$, $^{**}p < 0.01$, $^{***}p < 0.001$.

skew-symmetric matrix with $D(D+1)/2$ free parameters to be optimized. Note that the rotations can be redundant in the sense that $\boldsymbol{R}_{c_i,c_j}$ and $\boldsymbol{R}_{c_j,c_i}$ may cancel each other. We thus add a term that penalizes the norm of $\tilde{\boldsymbol{R}}_{c_i,c_j}$ to the loss function (Eq. 2; $\tilde{\boldsymbol{R}}_{c_i,c_j} = \boldsymbol{0}$ results in $\boldsymbol{R}_{c_i,c_j} = \mathbb{I}$).

bioRxiv preprint doi: https://doi.org/10.64898/2025.12.06.692615; this version posted December 11, 2025. The copyright holder for this preprint (which was not certified by peer review) is the author/funder, who has granted bioRxiv a license to display the preprint in perpetuity. It is made available under aCC-BY 4.0 International license.

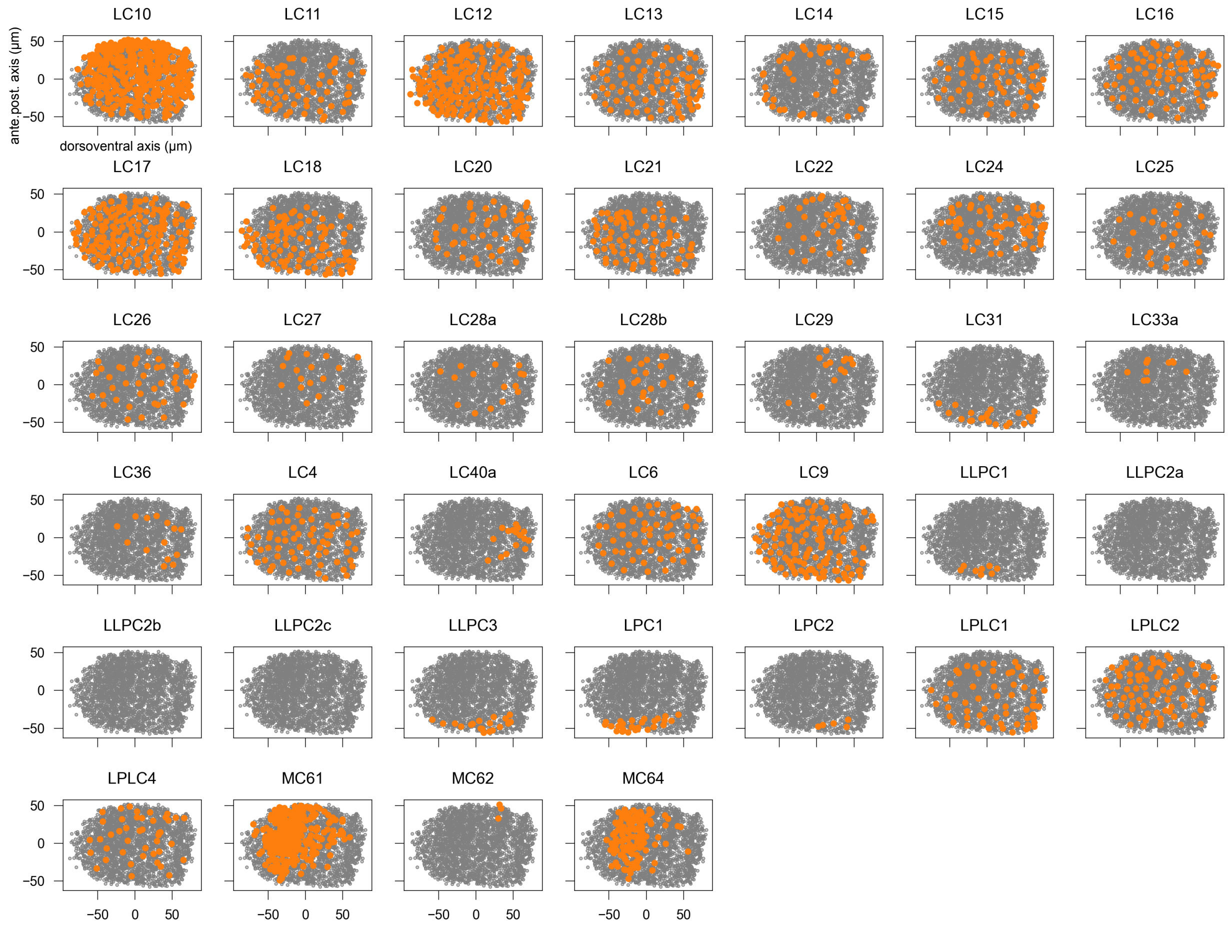

Figure S2: Estimated ground-truth retinotopy of columnar visual projection neurons. C.f. Fig. 6B. Each dot is the spatial centroid of optic-lobe synapses of a given neuron. Gray dots are all the cell types and orange dots are the given cell type. The centroids have been projected onto the top two PCs of all the centroids pooled together. See Fig. 6A.

bioRxiv preprint doi: https://doi.org/10.64898/2025.12.06.692615; this version posted December 11, 2025. The copyright holder for this preprint (which was not certified by peer review) is the author/funder, who has granted bioRxiv a license to display the preprint in perpetuity. It is made available under aCC-BY 4.0 International license.

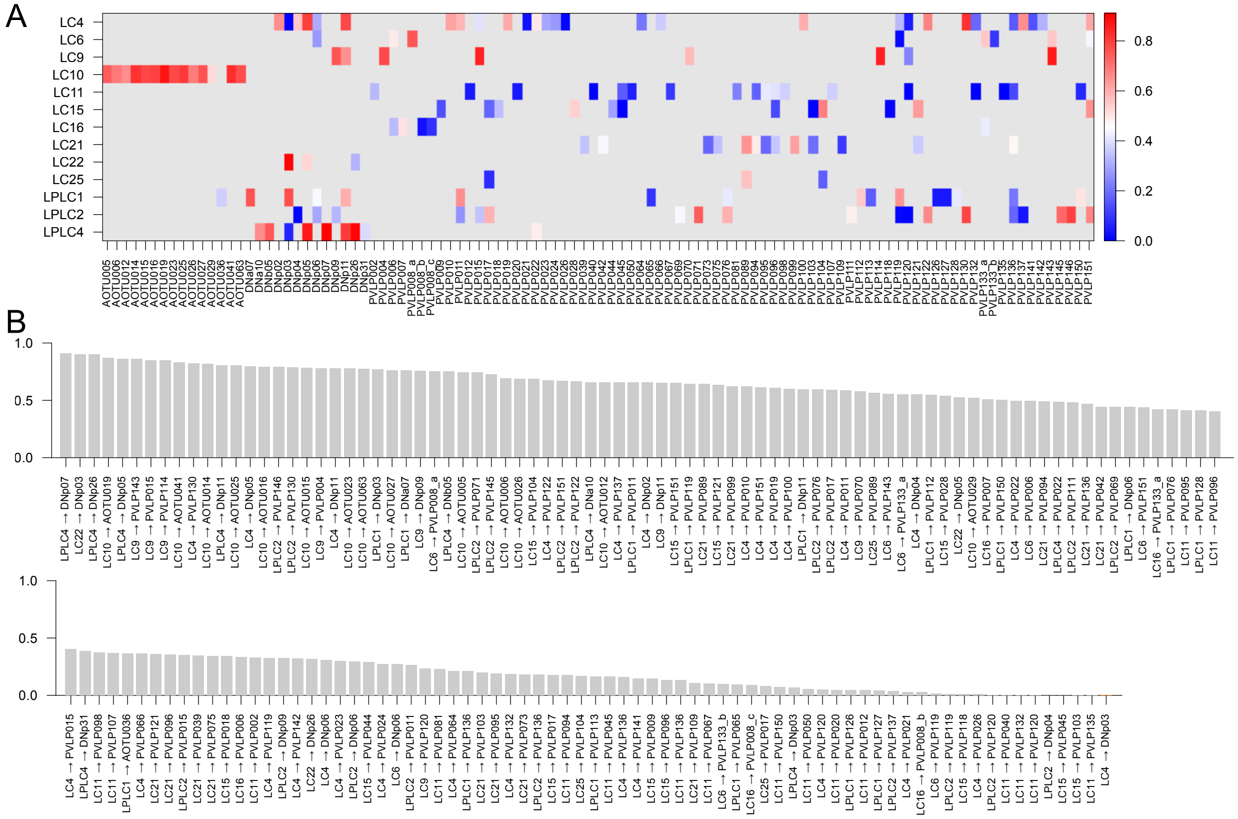

Figure S3: Model goodness-of-fit for projections from select VPNs to their target cell types. Same data as those presented in Fig. 6**F**,**G**. For each pair of cell types (types m, n), it is measured by computing the r^2 between the actual numbers of synapses from each VPN neuron to each target neuron across all pairs ($N_m \times N_n$) and those predicted by the fitted model. Projections between cell types with less than, on average, one synapse per neuron pair were excluded from further analysis (Methods 5.4) The VPN types selected are those with high retinotopy scores, as displayed in Fig. 6E.
